# Detecting Attention Levels in ADHD Children with a Video Game and the Measurement of Brain Activity with a Single-Channel BCI Headset

**DOI:** 10.3390/s21093221

**Published:** 2021-05-06

**Authors:** Almudena Serrano-Barroso, Roma Siugzdaite, Jaime Guerrero-Cubero, Alberto J. Molina-Cantero, Isabel M. Gomez-Gonzalez, Juan Carlos Lopez, Juan Pedro Vargas

**Affiliations:** 1 Department of Experimental Psychology, Faculty of Psychology, Universidad de Sevilla, 41018 Seville, Spain; aserrano3@us.es (A.S.-B.); jclopez@us.es (J.C.L.); 2 Department of Experimental Psychology, Faculty of Psychology and Educational Sciences, University of Ghent, 9000 Ghent, Belgium; romasiugzdaite@gmail.com; 3 Department of Electronic Technology, ETSI Computer Engineering, Universidad de Sevilla, 41018 Seville, Spain; jaimereben@gmail.com (J.G.-C.); almolina@us.es (A.J.M.-C.); igomez@us.es (I.M.G.-G.)

**Keywords:** attention, brain–computer interface (BCI), prevention, early detection, ADHD, neurofeedback

## Abstract

Attentional biomarkers in attention deficit hyperactivity disorder are difficult to detect using only behavioural testing. We explored whether attention measured by a low-cost EEG system might be helpful to detect a possible disorder at its earliest stages. The GokEvolution application was designed to train attention and to provide a measure to identify attentional problems in children early on. Attention changes registered with NeuroSky MindWave in combination with the CARAS-R psychological test were used to characterise the attentional profiles of 52 non-ADHD and 23 ADHD children aged 7 to 12 years old. The analyses revealed that the GokEvolution was valuable in measuring attention through its use of EEG–BCI technology. The ADHD group showed lower levels of attention and more variability in brain attentional responses when compared to the control group. The application was able to map the low attention profiles of the ADHD group when compared to the control group and could distinguish between participants who completed the task and those who did not. Therefore, this system could potentially be used in clinical settings as a screening tool for early detection of attentional traits in order to prevent their development.

## 1. Introduction

Attention deficit hyperactivity disorder (ADHD) is a multidimensional disorder characterised by a mixed pattern of symptoms that can be divided into two categories: (1) lack of attention and (2) hyperactivity plus impulsiveness. The latter includes behaviours such as a lack of attention to details, excessive talking, and excessive motor activity [1]. ADHD children display these characteristics early in their development, causing a negative impact on the social, emotional, and cognitive aspects of their normal development [2,3]. The prevalence of ADHD is estimated at approximately 5% for children, and this diagnosis persists in adulthood in 2.5 to 4.5% of cases [4]. ADHD includes inattention, hyperactivity, and impulsive subtypes that constitute separable but substantially correlated dimensions [5]. The criteria used by current diagnostics are based on symptoms, requiring the patient or their relatives to evaluate the frequency, intensity, and duration of symptoms. Due to the absence of biological markers of the disorder, the diagnosis might be subjective. In this regard, brain signal studies have been developed in order to overcome this issue and to achieve a diagnostic based on quantitative data [6,7].

Attention can be defined as the ability to focus continuously on a particular action, thought, or object. Several physiological markers can be used to indicate attention levels: regional cerebral blood flow, which increases during attentional tasks such as reading, naming, etc. [8]; pupil diameter [9] and blinking rate [10], which increase or decrease respectively as attention increases; and, finally, markers derived from electroencephalographic activity [11]. EEG studies have shown with accuracy several wave patterns associated with ADHD, detecting a high percentage of patients with attentional problems [11,12]. The main drawback of these techniques is that complex EEG–BCI systems require several electrodes placed on the scalp [13] and are not portable, which might be a problem if measures need to be done outside the lab. Furthermore, a traditional EEG has complex technical requirements, is costly, and is time-consuming to set up. Although in recent years there have been some attempts to use a simplified or low-cost version of an EEG for clinical purposes [14], the development of these technologies has risen exponentially for controlling devices [15,16]. Technology based on brain–computer interfaces (BCI) is recently being used to perform studies in natural contexts, which might likely be of practical value for clinical use in the near future. Some companies, such as Emotiv and NeuroSky, have released portable, wireless, consumer-oriented BCI headsets. Comparative research between both low-cost systems revealed that NeuroSky is more user-friendly and easier to set up and maintain [17]. These features are advantageous for clinical purposes, especially when it comes to preventing diseases or helping in diagnoses. The NeuroSky MindWave (NSMW) device has the potential to make the assessment of participants, especially children, more accessible and efficient. Additionally, the EEG signal recorded with the NSMW is comparable with a medical-grade EEG with limitations related to noise and spectral differences at low frequencies [18].

The NSMW has been developed as a non-invasive tool and involves placing a dry electrode on the left side of the frontal area corresponding to the Fp1 position. It provides information through a Bluetooth connection that can be classified according to three levels of processing [19]. From the lowest to higher levels, they are: (1) the raw EEG signal; (2) power bands (alpha, beta, theta, delta, and gamma); and (3) eSense brainwave patterns of attention and meditation. Power bands and eSense signals help to reduce the pre-processing of raw signals in external devices and allow the use of digital systems with low computation resources, also minimising the cost and time of the analysis in contrast to other technologies such as virtual reality (VR) [20]. Attention and meditation values are reported on a relative scale ranging from 1 to 100. The proprietary algorithms used to compute attention and meditation meters are based on both temporal and frequency domains. The exact algorithm has not been published, but the manufacturer asserts that the former has more emphasis on beta waves, whereas the latter has more emphasis on alpha bands. Several researchers have included the NSMW in their studies, showing the feasibility and reliability of using this technology in detecting or measuring emotional states [5,21,22], attention [23,24,25,26], and meditation states [25,27]. It has been shown that the NSMW provides accurate readings regarding attention as well as a positive correlation between the attention level reported on the device and the self-reported attention levels of the participants [26]. A similar study concluded that the NSMW accurately measured the overall level of mental attention [28]. Several studies have reported significant variations in the theta, alpha, and beta bands in the EEG recordings for several types of ADHD patients [29,30]. Therefore, the existence of a relationship between the attention indexes of NSMW and ADHD is highly probable.

Due to the lack of handy and attractive tools to evaluate attentional biomarkers in children, researchers have been developing games based on neurofeedback (NF) for training attention [31]. For instance, in an EEG study [32], two sets of electrodes were used to control the position of an object on a computer screen by means of attention. Results showed that a high percentage of participants (70%) in the experiment could control the game using only one electrode, which shows the feasibility of detecting attention with a reduced number of electrodes. Another study found that there is a significant improvement in children with ADHD when NF is included in their treatment [33]. The theta/beta ratio and slow cortical potentials (SCP) were combined as the control signal for NF. Moreover, two games were developed with the same NF control signals [34]. In one of them, a boy on a rope moved ahead if the participant had reduced theta activity and increased beta activity simultaneously for a period of time. In the other game, a ball moved upwards or downwards according to the participant’s SCP. With each successful attempt, a part of the covered picture became visible. Results showed that theta/beta training decreased the posterior midline theta activity, whereas the SCP training increased the central midline alpha activity. Both facts are associated with improvements in the German ADHD rating scale. Using the theta/beta ratio and electrical muscular activity as control signals in a game [35], children were trained in several tasks, such as keeping a ball above a cone. The training benefited ADHD children by improving their attention and reaction times. The results of these studies suggest that the attention indexes of NSMW are related to ADHD. In all these studies, the number of sessions needed to make the NF treatment effective was high. Here, we use an NF application game and the EEG­–BCI technology to obtain markers for ADHD with a low number of sessions, minimising the influence of the NF in the measure of the marker.

The present study analysed the attention parameter recorded from the cortex in a cohort of participants with and without an ADHD diagnosis. In order to evaluate possible attentional biomarkers between participants showing differences in attentional skills, we designed an application video game through GokEvolution. We tested sustained attention by analysing the EEG–BCI index provided by the NSMW device while participants played the GokEvolution game. This video game allowed us to catch the attention of children and to encourage a good performance while the clinical assessment process was completed.

## 2. Materials and Methods

### 2.1. Participants

In total, 52 control (32 boys, 20 girls, mean age 8.98 years, std 1.25) and 23 ADHD (18 boys, 5 girls, mean age 9.5 years, std 1.55) children were evaluated. All the participants had normal or corrected-normal vision. The study was approved by the Comité Coordinador de Ética de la Investigación Biomédica de Andalucía, Junta de Andalucía (Spain) with the code (1221-N-17). Participation in the study was voluntary, and participants gave informed consent to partake in the experimental procedure. A convenience random recruiting of control subjects was conducted for this study. ADHD was the only clinical condition with which the participants in the experimental group were diagnosed. None of the children with ADHD were on medication during the experiment. Children taking methylphenidate (MPH) had been off medication for 24 h, as the duration of MPH action ranges from 3–6 h for the immediate-release formulation, to 8–12 h for the extended-release alternatives [36]. No medication other than MPH was used for the subjects.

### 2.2. Clinical Instruments

The Test on Perception of Differences CARAS-R [37] was used to identify attentional skills as sustained and selective attention and impulsivity behaviour in children from 6 to 12 years old. The main task of the test is to identify differences between similar elements. We used it to analyse two measures of our interest: 1) effectiveness related to attention, and 2) impulsivity. Effectiveness in attention (AE) is based on the number of correct answers (A) and errors (E) made during the test. The AE index evaluates the participant’s performance during the test, penalised by the number of errors, which is subtracted from the number of correct answers. The impulsivity measurement is defined as the index of impulsivity ICI that indicates the proportion of effective and total performance. It is calculated by using this formula: ICI = (A − E)/(A + E) × 100%

The combination of the effectiveness and impulsivity variables expressed in enneatypes allowed us to classify the children into two groups: the effective and non-impulsive group, and the effective and impulsive group.

### 2.3. Design of GokEvolution Application Game

**Figure 1:**
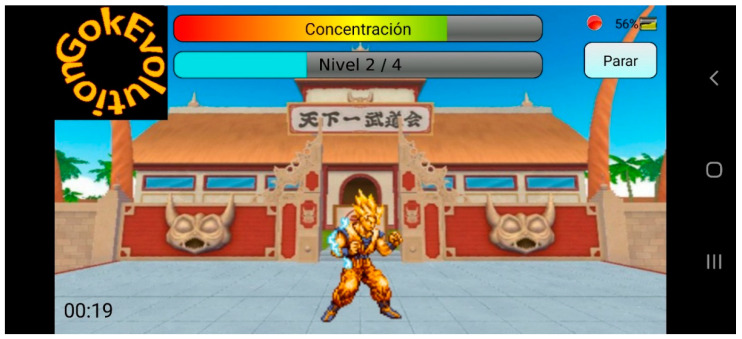
Screenshot of the GokEvolution application. The bars at the top indicate the level of attention recorded by the NeuroSky using the EEG sensor (top) and the achievement on the current level (bottom). The figure represents the character at level 2 (out of 4). If the attention level is higher than 50%, the character is “recharging energy” and the progress on the level increases. When level progress reaches the maximum (the whole bar) the game increases the level, changing the appearance of the character.

An attractive and colourful character was chosen in designing this application. We used evolutions of the character (different hair colour and shape) as markers of the level of the game (Figure 1). The game was originally designed as a neurofeedback software. The NSMW sends the attention data to the software, and to progress in the game the subject has to maintain a high level of attention. In a later stage, we defined the different levels and the parameters needed to progress through them in collaboration with clinical psychologists who specialised in children with ADHD. The game was codified under an Android format that could be downloaded on devices such as mobile phones or tablets from https://github.com/jaimegucu/EEGMindroidGokEvolution (accessed on 28 April 2021). It had simple instructions and a customisable level of difficulty.

### 2.4. Training Protocol for GokEvolution

The game had five difficulty levels increasing from level 0 to level 4 wherein the character appears with a different hair colour and shape, representing his evolution. The aim of the game is to achieve the complete evolution of the character through five difficulty levels. The main screen of the game shows the level of attention (NSMW index) and the accumulated points gained in each level (Table 1). During the five minutes the game lasted, the participant’s left frontal lobe activity was recorded using NSMW, which also recorded the attention and meditation parameters at a rate of 1 Hz. The attention level demanded by the game increased as the difficulty level did.

In each level, the character shows his evolution with a different hair colour and shape. To progress in the game, players had to gain points by paying as much attention as possible; otherwise, they could lose points. No other interaction with the game was available other than the EEG data from the NSMW, therefore, to progress in the game, players had to maintain an attentive state during play time. The level of the game determined how much time in an attentive state was needed to pass to the next level. The game considered that the player was in an attentive or inattentive state when the attention meter sent by NSMW ranged from 50 to 100, or from 0 to 50, respectively. According to the difficulty level, the number of points added/subtracted to/from a global score changed. Table 1 summarises the quantities used in each level. Please note that, as the difficulty level increased, a lesser number of points were added when in the attentive state, and a greater number of them were subtracted when inattentive. In order to continue to the following levels, the player had to accumulate a certain number of points that also changed according to the level. A player who managed to stay in an attentive state throughout the whole game would finish the five levels in 5 s, 9 s, 14 s, 20 s, and 70 s respectively. This means that it would take approximately 2 min to complete the evolution of the character with a perfect performance.

Importantly, players could monitor their performance in real time (neurofeedback) through the main screen of the game that shows two horizontal bars indicating the level of attention (NSMW meter) and the accumulated points gained in each level (Table 1). Both bars were scaled in a range between 0 and 100. Once the game was over, we obtained the average of attention per level (per participant).

### 2.5. Data analysis

A *t*-test was used to analyse differences in attention between the control and the ADHD groups per level. The significance level was set to 0.05, corrected for the multiple comparison with the Bonferroni correction for multiple tests. The levels were set from 0 to 4. As a final step, we correlated the NSMW indexes of attention with the behavioural measures, effectiveness and impulsivity, from the CARAS-R psychological test.

## 3. Results

### 3.1. GokEvolution Application Game and NeuroSky

We developed the GokEvolution EEG–BCI application game to test children with ADHD and those without. The goal of the game is to accumulate points by increasing the level of attention (NSMW parameter). Both parameters are displayed in the main screen during the game allowing players to monitor their performance in real time (NF).

**Figure 2:**
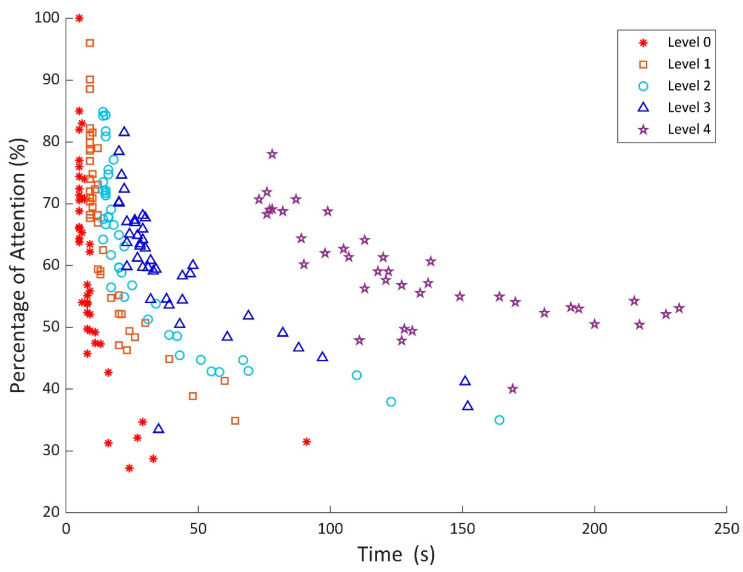
Mean attention values versus completion time for each level. As expected, these two variables follow an inverse relationship in each level.

**Figure 3:**
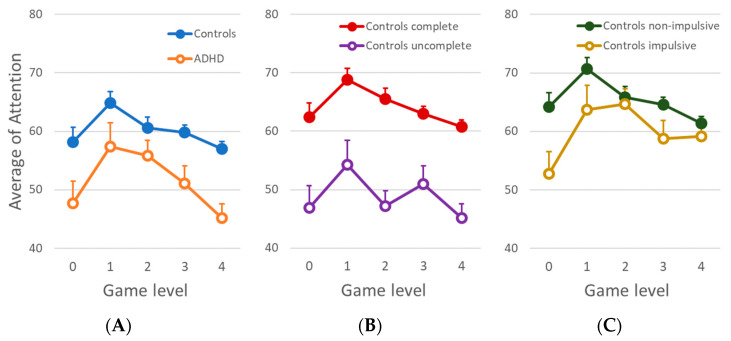
(**A**) Comparison of mean attention values between the ADHD and control group in each game level. (**B**) Comparison of mean attention values between controls that completed the five levels of the game and controls that did not complete the five levels of the game. (**C**) Comparison of mean attention values between controls that scored impulsivity at ICI index and non-impulsive controls.

According to the design of the present application, children were able to modulate their brain activity using the NF while they were playing the video game. Thus, we expected subjects with a high attentional level to complete the different phases of the game faster than subjects with a low attentional level. Additionally, the game was developed with the aim to increase attentional resources to proceed through the levels. Hence, the higher the level of the game, the more time it would take to complete the phase. In order to test our BCI application game in the control group, we plotted the attention average and the total time to complete a level (Figure 2). Results met the expectations described above; that is, a decrease in the percentage of success as the game level increased. To check for possible attentional differences between the control group and the ADHD group, we calculated the mean attention across all levels (Figure 3A). The ADHD group showed a lower and more variable average attention than the control group across all levels of the game. The differences between groups were significant in levels 0, 1, 3, and 4 (all ts(73) > 1.99; all ps < 0.05) but not in level 2 (t(73) =1.42, *p* = 0.160).

From the behavioural perspective, the inability to finish the game could be related to a lack of attention to the game, since the participants had to maintain high levels of attention during all levels in order to complete each stage. In this regard, level 4, the last level, was the most difficult one to complete, and not all participants overcame it. In order to analyse the attention parameter in this level, the control group was divided into two subgroups: participants that completed all five levels of the game, and participants that did not complete the last level of the game. We compared the output of the attentional level from the NSMW between these subgroups (Figure 3B) and found a different performance between both groups. We observed significantly higher attention scores in the group that completed all levels, including the levels finished by both groups. Statistical analyses displayed significant differences in all levels (all ts(50) > 3.32; all ps < 0.001). In addition, we found a better performance at the beginning of the game in the control classified as non-impulsive than those classified as impulsive, even though both groups showed a similar score in the rest of the levels.

### 3.2. Clinical Measurements

The clinical measurements, effectiveness (effective and ineffective) and impulsivity (impulsive and non-impulsive), were obtained from a CARAS-R test. We analysed whether effectiveness and impulsivity were associated with the completion of all levels. The subgroups were the same as in Section 3.1. We compared the performance in the game in the control group, divided according to the CARAS-R test in impulsive and non-impulsive profiles (Figure 3C). We found significant statistical differences in level 0 (t(36) = −2.10, *p* = 0.042) and level 3 (t(36) = −2.11, *p* = 0.042). In addition, there was a high variability in the scores of effectiveness and impulsivity in the ADHD group, in particular due to the fact that the groups were marginally reduced after the subdivision. The descriptive analysis of effectiveness and impulsivity of this subdivision is presented in Table 2.

## 4. Discussion

We developed a BCI application game in which the attention level, measured by NeuroSky, was monitored and used to complete levels of differing difficulty. The NSMW device proved to be sensitive to attentional changes while children played the GokEvolution application video game. Our preliminary results showed that attention measures given by the EEG–BCI device can be used as an attentional biomarker for the prevention of risk factors associated with attention diseases. We found higher attentional levels in the control group compared to the ADHD group, which would support the aforementioned lack of attention in children with an ADHD diagnosis. The key levels of the GokEvolution game for these differences were levels 0 and 4. Level 0 was the level where a larger number of children showed the highest involvement of attention in both groups, and it was significantly higher in the control group than in the ADHD group. Gradually, the number of participants able to progress through the game to the last level (level 4) decreased with each level. The reduced number of participants could be explained by the difficulty in keeping concentration for a long time. In fact, several participants did not finish the game.

The game allows for us to identify attentional profiles based on the subjects’ performance in the game. For instance, participants of the control group who completed all levels could be a sample of typically developed children, especially when we compare the subgroups divided by the impulsivity criteria based on the CARAS-R test (Figure 3C). Even though the impulsive group showed similar scores in levels 1, 2, and 4, they displayed a worse performance at the beginning. That is, even though they reached the goal, they did it with a lower score. On the other hand, we also found that the attentional level of the group that completed the game was significantly higher than the group that did not complete the game. This behaviour was observed in almost all levels of the game. We might explain the data by differences in sustained attention between groups over time. A better performance and completion of the game indicated an improvement in the control of attentional resources. That would mean that the group who could not complete the game outperformed the required scores of the first level of the video game application, but then could not maintain attention through to the final levels of the game. Moreover, we suggest that the subgroup of control subjects, who did not complete the last level, might have some problems regarding sustained attention. In any case, this setup is intended for a quick and massive screening of the population, therefore any possible disorder would then have to be adequately diagnosed using proper clinical methodology.

Our study provides a preliminary framework that could help identify biological markers of possible attentional problems and define specific endophenotypes. The development of endophenotypes might be more adequate for the effective application of pharmacological and behavioural treatments than traditional classifications of mental diseases based on diagnosis scales [38]. The evidence from this study suggests that the application videogame, in combination with the NSMW, could work as a pre-screening diagnosis tool to detect disorders related to attention. Even though our study only used one electrode (Fp1, more limited than a multichannel EEG device), we were able to measure attention in a non-clinical environment, which holds potential for data accuracy.

The EEG–BCI video game preliminary results showed that the attention variability could be used as an attentional biomarker that might have implications for early detection of traits associated with inattention. The question we raised in this study was whether we can correctly differentiate clinical cases using NSMW measurements while children play a GokEvolution video game. We were able to detect the variability differences between groups that completed and did not complete the game. Nevertheless, importantly, we were able to distinguish groups that are in the limit zone or at the borderline between ADHD and control attentional profiles.

The strength of the present study could increase the impact of digital tools used in a clinical setting, allowing us to use this proposed biomarker for prevention purposes and, if needed, in early detection for a more accurate behavioural and pharmacological treatment. The fact that GokEvolution was developed to be used on a tablet device increases usability of the training program. This video game application is fast to run, since it takes in total a maximum of 15 min to prepare the entire setup and to carry out testing testing, and is easy to perform while being fun for children. Another major advantage is the fact that the training can also be carried out independently at hospitals, private centres, or schools. The application can be used everywhere; there is no need for an internet connection during training. Finally, the validation of the test could be adjusted for age ranges in the future. Although our experimental setup consisted of an NSMW combined with a neurofeedback game designed by our group, similar systems could be used instead of the one proposed in the present study. More sophisticated low-cost EEG systems could be more adequate for a clinical setup at the expense of a more limited usability [14].

Taken as a whole, we propose that levels of attention and behavioural measures could help us characterise our participants in different populations. Studies carried out by our research group have shown a clear correlation between attention deficit and levels of impulsivity in an animal model [39]. These endophenotypes are based in different cue processing, predict vulnerability to behavioural disorders [40], and could work as a model to evaluate individual differences regarding impulsivity and attention factors [7].

## 5. Conclusions

Our research underlined the importance of objective tests to evaluate features in early detection for the prevention of risk factors in cognitive disorders. This new generation of video games, in combination with behavioural tests, could be used to evaluate possible risk factors in order to prevent the development of attention related disorders. Although these results need to be replicated to achieve definitive conclusions, the development of digital tools might support our knowledge of clinical interpretation, establishing a more appropriate evaluation and pharmacological and behavioural therapy approach.

## Figures and Tables

**Table 1:** Values added/subtracted to global score in each level of the game according to the level of attention.

Level	0	1	2	3	4
Attentive	+18	+16	+14	+12	+10
Inattentive	−2	−3	−4	−5	−6

**Table 2:** Effectiveness and impulsivity in the subdivision of the ADHD and control groups in the completed and uncomplete level.

	Effective	Ineffective	Impulsive	Non-impulsive	TOTAL
ADHD completed	3	7	9	1	10
ADHD uncompleted	8	5	7	6	13
Total	11	12	16	7	23
Controls completed	38	0	9	29	38
Controls uncompleted	13	1	4	10	14
Total	51	1	13	39	52

## Data Availability

Data are contained within the article.
